# Biogas Cook Stoves for Healthy and Sustainable Diets? A Case Study in Southern India

**DOI:** 10.3389/fnut.2015.00028

**Published:** 2015-09-16

**Authors:** Tal Lee Anderman, Ruth S. DeFries, Stephen A. Wood, Roseline Remans, Richie Ahuja, Shujayath E. Ulla

**Affiliations:** ^1^Environmental Defense Fund, San Francisco, CA, USA; ^2^Department of Ecology, Evolution, and Environmental Biology, Columbia University, New York, NY, USA; ^3^Agriculture and Food Security Center, The Earth Institute, Columbia University, New York, NY, USA; ^4^Bioversity International, Addis Ababa, Ethiopia; ^5^Department of Social Work, St. Joseph’s College, Bangalore, India

**Keywords:** alternative cook stove, biogas cook stove, diet diversity, nutrition, time allocation, time savings, India

## Abstract

Alternative cook stoves that replace solid fuels with cleaner energy sources, such as biogas, are gaining popularity in low-income settings across Asia, Africa, and South America. Published research on these technologies focuses on their potential to reduce indoor air pollution and improve respiratory health. Effects on other cooking-related aspects, such as diets and women’s time management, are less understood. In this study, in southern India, we investigate if using biogas cook stoves alters household diets and women’s time management. We compare treatment households who are supplied with a biogas cook stove with comparison households who do not have access to these stoves, while controlling for several socio-economic factors. We find that diets of treatment households are more diverse than diets of comparison households. In addition, women from treatment households spend on average 40 min less cooking and 70 min less collecting firewood per day than women in comparison households. This study illustrates that alongside known benefits for respiratory health, using alternative cook stoves may benefit household diets and free up women’s time. To inform development investments and ensure these co-benefits, we argue that multiple dimensions of sustainability should be considered in evaluating the impact of alternative cook stoves.

## Introduction

Alternative cook stoves are becoming increasingly popular in low-income settings across Asia, Africa, and South America (1). These stoves replace solid fuels, such as wood, animal dung, and crop residue, with cleaner energy sources such as biogas. Yet an estimated 2.6 billion people still cook with solid fuels, with more than 95% of them in Asia and Sub-Saharan Africa (1). Household air pollution from openly combusting solid fuels indoors has been cited as the third largest risk of premature mortality, responsible for more than 108 million disability-adjusted life-years (2–5). Reducing usage of solid fuels through, for example, biogas cook stoves is therefore crucial to avoid the more than 4.3 million deaths attributed to household indoor air pollution annually (2, 3, 6).

Serious health consequences are an important reason to address the challenge of transitioning households away from openly combusting solid fuels. Yet many economic, technological, and cultural obstacles can deter households from making these changes including, for example, a lack of affordable alternative energy options and cultural preferences for traditional fuel sources (4, 7–9).

In recent years, there has been a surge in efforts to promote alternative cook stoves (10, 11). These efforts include replacing traditional solid cooking fuels with cleaner energy sources, such as liquid petroleum gas (LPG) and biogas. Other options include rocket stoves, which improve heat-use efficiency thereby reducing the amount of fuel required, and gasifier stoves, which more thoroughly combust particulate matter to reduce net emissions (12). Organizations such as the Global Alliance for Clean Cookstoves, which has almost 400 partners in 36 countries, support the financing and deployment of these technologies.

The main body of existing literature focuses on the effect of alternative cook stoves on respiratory health. Evidence shows that using alternative cook stoves significantly reduces indoor air pollution, including concentrations of particulate matter and carbon monoxide (13–17). A plethora of studies illustrate the link between reductions in indoor air pollution and improved respiratory health (18–24). Additional research provides insights on best practices for the distribution and promotion of alternative cook stoves by carefully considering cultural, physical, and economic barriers to adoption (4, 7, 8, 25–29).

Taking together the existing literature, there is a clear knowledge gap in how changing cooking technologies influences other aspects related to cooking, such as dietary patterns and women’s time management. In nutrition science, diet diversity is well established as a key component of a quality diet (30–34). Studies have shown that higher individual diet diversity relates to higher micronutrient intake for children (35–38), adolescents (39), and adults (30, 31, 40). Household diet diversity is used as an indicator for household access to a variety of foods (41).

We hypothesize that changing the type of cook stove a household uses could trigger shifts in the diversity of foods they consume (42). For example, because cooks have more control over temperature and cooking time with an alternative stove, they might choose to add different items to their meals that were previously too time-consuming or posed too high a spoilage risk because of uncontrollable stove temperatures. Similarly, families may have avoided preparing foods with longer cooking times, such as certain legumes, to save fuel wood, or to avoid creating indoor air pollution. Changing cooking technologies may also shift household expenditures and time management, allowing greater access to a diversity of foods.

Similar to the effects on diet diversity, little research has investigated how households manage their time depending on the cooking technology they use (43, 44). We hypothesize that because alternative cook stoves require less or no wood, the use of these stoves reduces the amount of time that households, particularly women, need to spend on firewood collection. In addition, the time required to cook could also be reduced because of better heat control. We hypothesize that such time-savings can free up time for other activities, such as income generation or relaxation.

To test these two hypotheses – the effect of alternative cook stoves on diet diversity and women’s time management – we use a case study of biogas cook stoves in southern India.

Biogas cook stoves are fueled through anaerobic digestion of dung, which takes place in an anaerobic digester belowground (45). One product of this digestion (methane) is piped to the house to fuel a cook stove, while the remaining content (known as slurry) flows out of the digester and can be used as fertilizer (45).

India is an important case study because roughly 70% of the population lives in rural communities and more than 75% of those rural households (over 750 million people) rely on traditional solid fuels for cooking (3, 11, 46, 47). In these regions, household air pollution is responsible for over 550,000 premature deaths of poor women and children each year (6, 10, 48, 49). The country has employed a variety of strategies to address this significant public health risk, including subsidizing biogas cook stoves (10, 11). At present, there are many biogas cook stove development projects operating throughout India (10, 11). However, to our knowledge, this is the first study that looks into the effect of these biogas cook stoves on diet diversity and time allocation.

## Materials and Methods

### Ethics statement

This study has been reviewed and approved by the Chair of the Columbia University Institutional Review Board-Morningside (IRB Protocol #AAAK9905). Following IRB-approved directions for this study, each head of household that participated provided voluntary informed consent, either in writing or through authorization of a portrait photograph taken of them by an enumerator.

### Study area and sampling design

**Figure 1:**
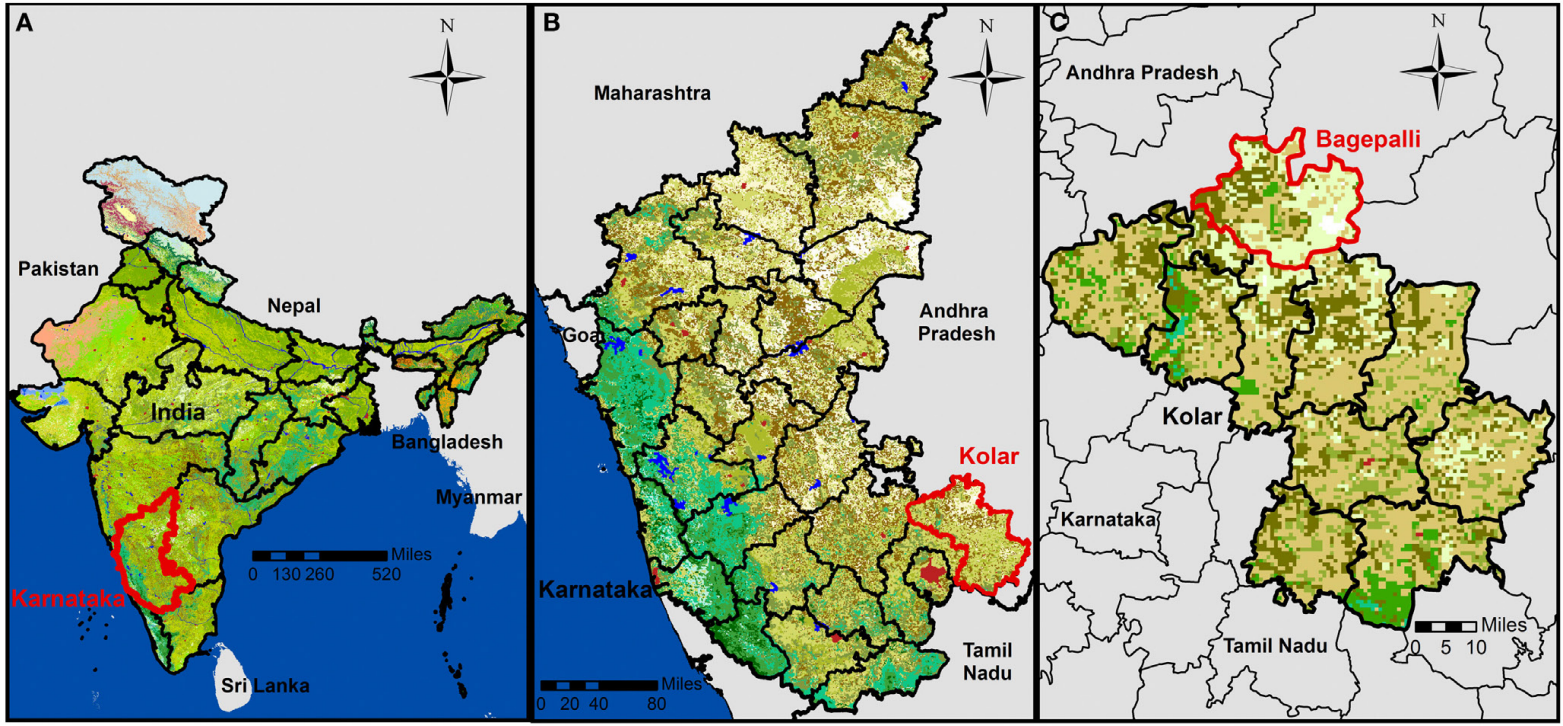
**Map depicting (A) the study area in the state of Karnataka, India; (B) in the district of Kolar; (C) in the Bagepalli *panchayat***. Created in ArcGIS using external data (50–53).

We analyzed household-level data for members of the Agricultural Development and Training Society (ADATS) in the state of Karnataka in southern India. ADATS is a membership-based organization of smallholder households that pool capital to address financial needs. Operating out of the town of Bagepalli, 100 km north of Bangalore, ADATS has about 30,000 participating families in over 1,000 villages in five *panchayats* (Indian self-governments at the village level) in the Kolar district of Karnataka (Figure 1) (50, 51). ADATS’ work spans multiple sectors, including adult literacy, alternative energy development, agriculture, child education, public health, legal aid, and mitigation of climate change.

**Figure 2:**
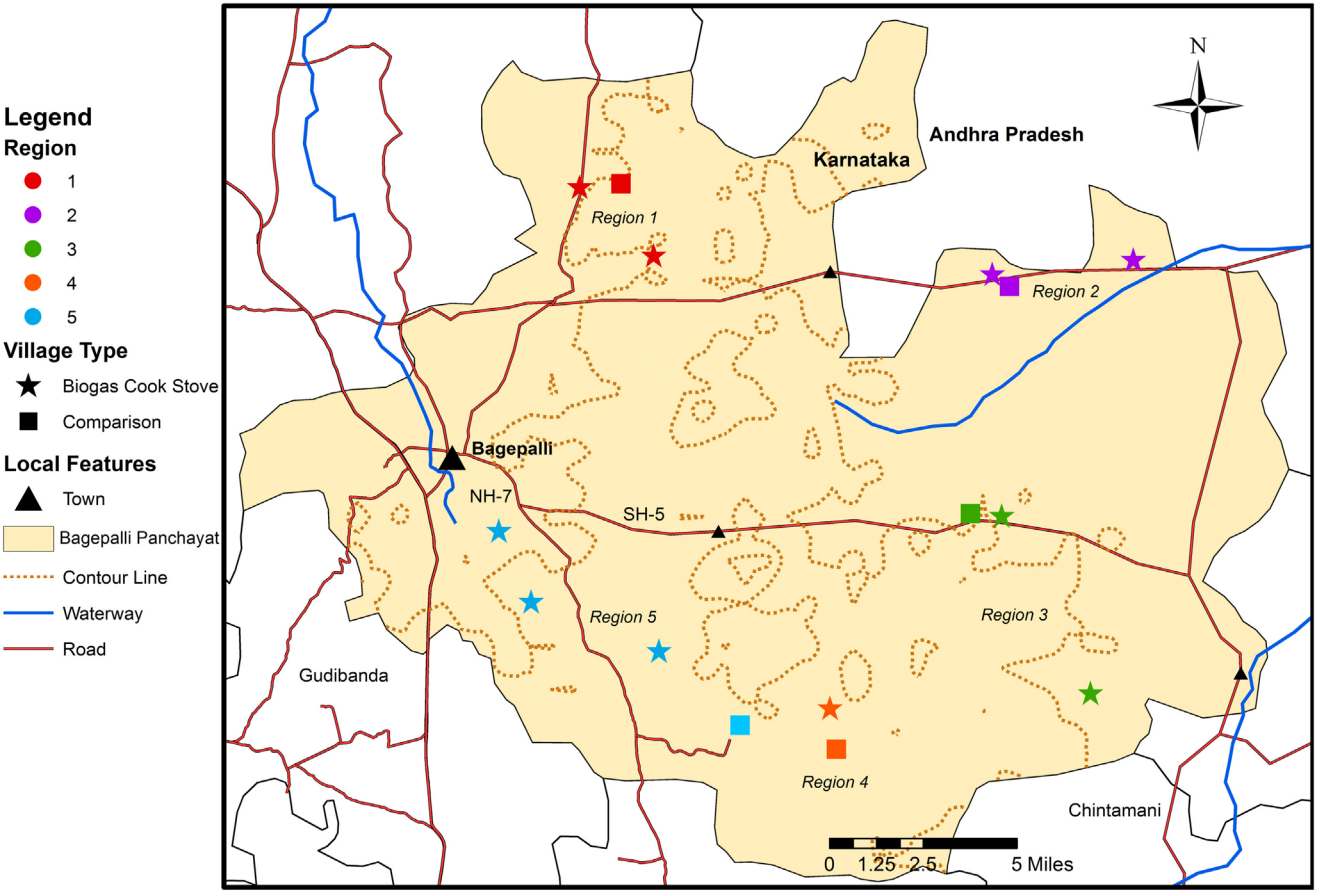
**Map illustrating the distribution of the study’s 10 villages with biogas cook stoves and five villages used as comparisons**. Created in ArcGIS using external data (50–53).

We surveyed 199 households in 15 ADATS member villages (Figure 2; Table S1 in Supplementary Material) from the Bagepalli *panchayat* in the Kolar district of Karnataka (Figure 1) (51–53). The Bagepalli *panchayat* skirts the southern border of the Rayalaseema desert. The terrain is semi-arid and drought prone, with rainfall averaging 560 mm/year (54). The majority of families in the region are labor workers, while a few tend their own agricultural plots.

Data from households using biogas cook stoves (the “treatment” group) were collected for families in 10 villages participating in the Bagepalli clean development mechanism (CDM) Biogas Programme. Half of the households in each selected village were randomly chosen for surveying using a random number generator. The Programme is an ADATS project launched in 2006 that built 5,500 biogas cook stove units in 128 villages. All ADATS member households in villages that could run a biogas cook stove – meaning that they had a cow, yard space, and the ability to dig a deep hole for the anaerobic digester – were given the option to obtain a stove. All stove materials were provided for free by ADATS.

A second set of five villages in the Bagepalli *panchayat* that did not have biogas cook stoves (but were still part of ADATS) were also surveyed to provide reference data (the “comparison” group). Due to disparities between the number of treatment and comparison households, all eligible households in comparison villages were surveyed. Comparison villages were chosen based on (1) their having been unable to dig the hole needed to construct the digester for the biogas cook stoves because of large subsurface rocks, (2) their physical proximity to the 10 randomly selected treatment villages, (3) their participation in ADATS, therefore receiving other wide-ranging benefits of membership, and (4) their having households that owned a cow and had adequate yard space and would have thus been able to run a stove when the Biogas Programme was launched in 2006, and when surveys were enumerated in 2013.

All households without a biogas cook stove (the comparison group) used a wood-burning stove inside the house to cook. Some households used multiple cook stoves. Five comparison households used a kerosene stove. No other alternative cook stoves were used in the comparison or treatment groups. Two treatment households used a firewood stove alongside their biogas unit. Whether a household owned a biogas, firewood, and/or kerosene stove is included in each of our models.

### Data collection and indicators

Data for this study (summarized in Table 1; available in Supplementary Material) were gathered from January to March 2013. A survey (Supplementary Material) was administered to 199 households, with the female household head acting as the main respondent. Of the 10 villages with biogas cook stoves, 141 households were surveyed. Fifty-eight households were surveyed from the five comparison villages. We used several metrics to assess the relationship between ownership of a biogas cook stove and co-benefits for household diet diversity and time savings (Table 1).

**Table 1:** **Synthesis of study variable characteristics, including variables for household dietary patterns, women’s time allocations, and a set of potentially confounding factors**.

Variable	Description	Range	Mean			Sample size (treatment/comparison)
			All	Treatment	Comparison	
**Household dietary patterns**						

Daily diet diversity score	Summed score on daily basis using 10 food groups	0–10	6.1 (1.0)	6.3 (1.0)	5.6 (0.9)	(141/58)

Weekly diet diversity score	Summed score on weekly basis using 10 food groups	0–10	9.2 (0.9)	9.3 (0.8)	8.9 (1.0)	(141/58)

Daily food variety score	Summed score on daily basis using 36 food items	0–36	9.1 (2.4)	9.8 (2.4)	7.6 (1.6)	(141/58)

Weekly food variety score	Summed score on weekly basis using 36 food items	0–36	20.8 (4.7)	22.1 (4.3)	17.7 (4.3)	(141/58)

Minimum diet diversity	If household consumes greater than five food groups daily	0 or 1	1.0 (0.2)	1.0 (0.1)	0.9 (0.2)	(141/58)

**Women’s time allocations**						

Cooking	Hours/day cooking and preparing food/snacks	0–7	2.9 (1.2)	2.7 (1.2)	3.4 (0.9)	(141/58)

Housework	Hours/day doing housework or yard work	0–10	2.4 (1.3)	2.4 (1.4)	2.5 (0.8)	(141/58)

Labor work	Hours/day engaged in paid labor work	0–10	6.5 (3.5)	6.2 (3.7)	7.2 (2.8)	(141/58)

Collecting firewood	Hours/day spent collecting firewood	0–4	0.4 (0.8)	0.0 (0.1)	1.2 (0.9)	(141/58)

Relaxing	Hours/day spent relaxing	0–5	1.3 (0.8)	1.3 (0.9)	1.1 (0.7)	(141/58)

**Potentially confounding factors**						

Kerosene	Household uses kerosene stove	0 or 1	n/a	0.0 (0.0)	0.1 (0.3)	(141/58)

Additional firewood stove	Treatment household uses firewood and biogas stove	0 or 1	n/a	0.0 (0.1)	0.0 (0.0)	(141/58)

Caste	Lower caste Upper caste	n/a	49% 51%	39% 61%	67% 33%	(105/57)

Religion	Hindu	n/a	89%	86%	97%	(141/58)
	Muslim		11%	14%	3%	

Years membership in ADATS	# years household a member of ADATS	3–34	13.3 (8.6)	12.1 (7.6)	15.6 (9.9)	(105/57)

Asset index	Wealth indicator (PCA of 20 assets)	−2.9−7.4	0.2 (2.0)	0.9 (2.0)	−1.0 (1.2)	(102/55)

Distance to major market	Kilometer from village to major market	7.8–37.8	22.0 (9.5)	21.6 (11.0)	23.2 (3.9)	(141/58)

Household size	Number of people living on same property	1–15	5.0 (2.4)	5.0 (2.5)	4.9 (2.0)	(141/58)

Dependency ratio	# Dependents/# Providers	0–2	0.4 (0.4)	0.3 (0.4)	0.4 (0.4)	(136/57)

*Table includes variable description, range, mean (all, treatment, and comparison households), and sample size. SDs are reported in parentheses for mean values*.

To generate a diet diversity score, which measures the variety of food groups consumed over a given time period, we asked the female household head to report on the household’s diet using a semi-quantitative food frequency survey. This included reporting the frequency (times per day, week, month, or year) that the household consumed 36 locally available food items (Tables S2 and S3 in Supplementary Material) (41, 55).

These foods were categorized into 10 food groups following the women’s diet diversity score as outlined by the Food and Nutrition Technical Assistance III Project (FANTA) and the Food and Agriculture Organization of the United Nations (FAO) (Table S2 in Supplementary Material): starchy staples, beans and peas, nuts and seeds, dairy, flesh foods, eggs, Vitamin A-rich dark green leafy vegetables, other Vitamin A-rich fruits and vegetables, other vegetables, and other fruit (55). Each food item received a value based on how often it was reportedly consumed (0 = never; 1 = once a week; 7 = once a day, and all other values scaled accordingly). These values were then summed across food groups for each household. By applying cut-offs in one-point increments, we established diet diversity scores that reflect the number of food groups – ranging from 0 to 10 – that households consumed on a daily (Day Diet Diversity Score) and weekly (Week Diet Diversity Score) basis.

The reported frequency of consuming the food items categorized into food groups for the diet diversity score were also used to generate a food variety score. The food variety score gives equal weight to the consumption of each of the 36 food items by summing the consumption frequencies of all items, rather than dividing the items into groups (41). The food variety score ranges from 0 to 36 food items consumed on a daily (Day Food Variety Score) and weekly (Week Food Variety Score) basis. The food variety score is also a proxy for diet quality (37). It complements the diet diversity score by offering insight into the number of different food items accessible, thus also providing a measure of household resilience. Because it has greater variation than the diet diversity score, it is more sensitive to differences between households.

The daily diet diversity score was also used to categorize diets according to a threshold suggested by FAO and FANTA as the women’s minimum diet diversity score (55). This yes/no indicator, in comparison to the range of values in the diet diversity and food variety scores, reflects if households have reached a minimum diet diversity threshold by consuming at least 5 out of 10 food groups on a daily basis (55). FAO and FANTA established the women’s minimum diet diversity score to reflect the finding that women consuming foods from five or more of the 10 food groups in the diet diversity score have a greater likelihood of meeting their micronutrient needs than women consuming foods from fewer food groups (55, 56).

Our second set of response variables quantified how much time female heads of households spent cooking, doing housework, doing paid labor work, collecting firewood, and relaxing during a workday. By having the female household head (respondent) map out her daily activities via a 24-h recall, we calculated the time she spent each day on these five activities. Each activity was analyzed independently.

A third set of data, including demographic, cultural, and socio-economic variables, was collected and used to account for other potentially confounding factors in our analysis. A household’s total size and its’ dependency ratio indicated household demographics. Household size was reported as the aggregate number of people dwelling on one property. The dependency ratio divided the number of household members between 0 and 14 and over 65 years of age by the number between 15 and 64 years of age (57). A larger value indicates higher dependency within the household. Cultural characteristics were represented by binary variables for a household’s religion (Hindu or Muslim) and caste. A range of the 14 castes, including Muslims, present in our study were grouped into upper and lower castes in consultation with local authorities.

Socio-economic variables were represented by a household’s duration of membership in ADATS, distance to a major market, and wealth. The distance on navigable roads between each village and the area’s major market was calculated using Google Maps (58). Wealth was measured using an asset index, which aggregates household stocks with different units (e.g., livestock, landholdings, and household appliances) to generate a wealth ranking between households in a study population (59–61). Many development economists now advocate the use of household welfare measures based on assets to assess poverty, given fewer biases related to respondent recall errors, survey seasonality, and measurement error (60). We collected data on household reported ownership of 20 asset indicators (Table S4 in Supplementary Material) (59). We then used a principal component analysis (PCA) to generate weights for each of the 20 assets. The first principal component in the analysis is the linear combination of the assets that explains the greatest sample variance in the data. The index itself is a summation of asset ownership, weighted by each asset’s contribution to the explanation of total variance relative to the first principal component (61). The asset index serves as a comparative measure of poverty; each household’s poverty ranking is relative to the study population used in the PCA (60). Research on the relative merit of different asset indices, including a metric of structured income, shows that the PCA-based asset index used here offers the best indication of the relative socio-economic position of each household, and therefore of local wealth distributions and orderings, in a study population (59, 60).

After initial surveys, a basic qualitative module was performed to enhance our understanding of community perceptions on household diet diversity, women’s allocations of time, and any other changes respondents experienced in association with owning a biogas cook stove (62–67). The informal focus group discussions were conducted in March 2013 in two villages with biogas cook stoves that were randomly selected from the 10 treatment villages. Through this random selection, the focus groups were held with the female household heads of surveyed households in one village in Region 1 and one village in Region 5 (see Figure 2). Because of the informal nature and structure of the focus groups, these discussions were only used to gain feedback on the preliminary trends and results found in the quantitative analysis, and to give back to the community by informing them about the expected outcomes of the research.

### Analysis

All statistical analyses were conducted in STATA (version 13) (68). Separate mixed models with fixed effects were run for each of the response variables. The 15 villages surveyed were grouped into five regions based on the geographic distribution of the villages, with two to three biogas cook stove and one comparison village per region (Figure 2). Individual villages were also applied as a grouping factor. However, the limitations in sample size per village made it difficult to interpret results from this grouping, which was therefore not used in the final analysis. Because comparison villages were intentionally selected based on requirements, such as geographic location and an inability to construct biogas cook stoves, a mixed model with fixed effects grouped by region was applied to account for possible non-random undocumented socio-economic or spatial village-level grouping effects. To further account for potential non-random effects, we applied robust clustering of standard error at the village level. This lowered the residuals in each of our models, measured through root mean square error (RMSE).

Throughout our analysis, continuous predictor variables were standardized by two standard deviations (SDs), binary predictor variables were centered, and response variables were left unstandardized (69). This standardization procedure was used to ensure that variables were expressed in common units so that correlation coefficients within each model could be compared. The data were tested for outliers using Cook’s Distance. Cook’s Distance was calculated for each model and, using the standard cut-off value of 1 (70), did not identify any outliers. Collinearity between variables was systematically checked using variance inflation factors (VIF); results indicated that there were no instances of collinearity as outlined in *Results*.

To add robustness to testing the assumption that our study population was analogous despite natural variations in household characteristics, we also ran the analysis on a subset of the population selected using propensity score matching (PSM). PSM is one approach to tackle the possibility of selection bias in choosing a treatment population. It addresses this issue by identifying a subset of the comparison group that is similar to the treatment group in all relevant pretreatment characteristics. Within this population subset, then, differences in outcomes between the comparison and treatment groups can be more easily attributed to the treatment itself (71, 72). We used a probit regression model to calculate the propensity score for each household, which estimates the probability of a household participating in the treatment given its observed covariates (72, 73). We included the following equally weighted covariates to calculate the propensity score: asset index, caste, religion, household size, dependency ratio, and duration of membership in ADATS (72, 73). When applying this structure to our data, the balancing property was satisfied. We then identified the population with a region of common support across all covariates by removing households with a propensity score smaller than the minimum or larger than the maximum of the opposite group (71, 72). This selected 19 households (13 comparison and 6 treatment households) that fell outside the region of common support and were thus removed for the supplementary analysis, bringing the population subset to 138 households out of the original 157. All models were run on this population subset as described in Section “Results.”

## Results

### Description of household characteristics

The 199 households in 15 villages analyzed in this study present a range of socio-economic, cultural, and geographic characteristics (Table 1; Table S1 in Supplementary Material).

### Hypothesis 1: Households with biogas cook stoves have a more diverse diet as compared to households without the stoves

To test our first hypothesis, we assess the relationship between household diet diversity and ownership of a biogas cook stove using a set of mixed models with fixed effects, which allows us to control for various socio-economic variables (Table 2). We consistently find a significant relationship between diet diversity and cook stove technology, independent of the type of diversity metric used (daily or weekly diet diversity or food variety score) – households that use a biogas cook stove have a more diverse diet than households that use a firewood stove.

**Table 2:** **Mixed model with fixed effects grouped by region for the daily and weekly diet diversity score and food variety score, and the minimum diet diversity score**. Biogas cook stove ownership reported as a binary variable with comparison households (0) and treatment households (1).

Variables	(1) Day diet diversity score	(2) Week diet diversity score	(3) Day food variety score	(4) Week food variety score	(5) Minimum diet diversity
Firewood_biogas	0.424*** (0.098)	0.361* (0.152)	1.523*** (0.247)	3.380*** (0.840)	0.005 (0.022)
Kerosene	0.633*** (0.056)	0.533 (0.375)	1.131* (0.486)	−0.370 (1.179)	0.033 (0.032)
Add_FIrewood	0.204 (0.201)	−1.091** (0.340)	−0.589 (0.288)	−6.803*** (1.174)	0.015 (0.032)
Asset_index	0.526** (0.144)	0.313 (0.173)	1.353** (0.390)	1.147 (1.116)	0.071 (0.048)
Caste	0.268 (0.161)	0.073 (0.121)	0.502 (0.240)	−0.009 (0.620)	0.008 (0.043)
Religion	−0.126 (0.459)	0.209 (0.205)	−0.593 (0.588)	1.727* (0.741)	0.018 (0.015)
Dist_market	0.274 (0.149)	−0.285 (0.346)	1.031** (0.301)	−0.622 (1.176)	−0.150** (0.040)
HH_size	0.245 (0.169)	−0.059 (0.095)	0.571 (0.438)	−0.264 (0.697)	0.023 (0.026)
Age_ADATS	0.228 (0.206)	0.013 (0.200)	0.470 (0.355)	−0.026 (1.260)	0.058 (0.041)
Observations	157	157	157	157	157
Number of region	5	5	5	5	5
Adjusted *R*-squared	0.159	0.079	0.416	0.274	0.046
RMSE	0.904	0.804	2.115	3.942	0.170

*Robust standard errors (SEs) are in parentheses*.

*** < 0.001, ** < 0.01, * < 0.05.

**Figure 3:**
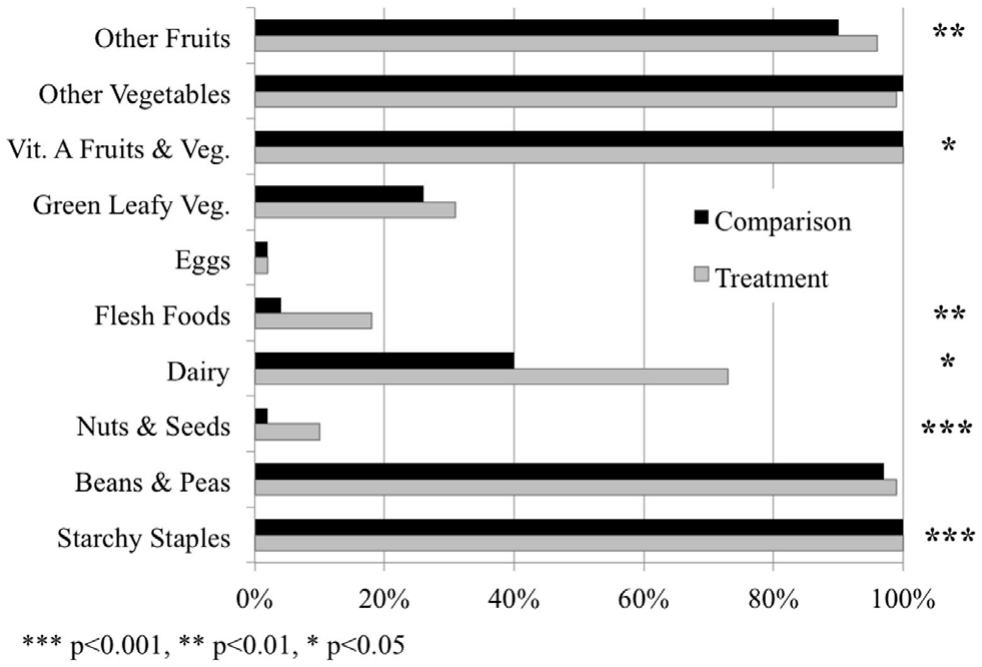
**The percentage of households consuming each of the 10 food groups in the diet diversity score on a daily basis in the treatment and comparison populations, respectively**. Significant differences in the consumption of food groups between the two populations represented by asterisks, based on analyses from mixed models with fixed effects.

One particular food item or food group does not explain the differences observed in diet diversity between treatment and comparison households. Instead, the difference is related to the treatment households’ higher average consumption of several food groups. Starchy staples, beans and peas, Vitamin A fruits and vegetables, and other vegetables are the most frequently consumed food groups in both populations (Figure 3; Table S3 in Supplementary Material). However, treatment households consume the following food groups significantly more frequently on a daily basis: starchy staples, nuts and seeds, dairy, flesh foods, Vitamin A fruits and vegetables, and other fruits (Figure 3; Table S3 in Supplementary Material). In the context of the local situation, where nuts and seeds, flesh foods, green leafy vegetables, and eggs are among the least consumed items in general (Table S3 in Supplementary Material), this higher consumption of a variety of food groups by households with a biogas cook stove can add important nutritional diversity to the diet.

For the minimum diet diversity score, there was not a significant difference between the proportion of treatment and comparison households meeting the diet diversity cut-off of consuming five food groups daily. We do, however, observe that a household’s distance to a major market is a significant determining factor in reaching the diversity cut-off; households that were further from a major market less frequently consumed five or more of the 10 food groups each day (Table 2). This finding is consistent with other recent studies (74).

The analysis did not experience problems with collinearity (all VIF values <1.23). Furthermore, all models were run on the data subset identified as comparable through PSM, and results were consistent with the full sample analysis (Table S5 in Supplementary Material).

### Hypothesis 2: Women in households with biogas cook stoves have different time allocations than women without the stoves

**Figure 4:**
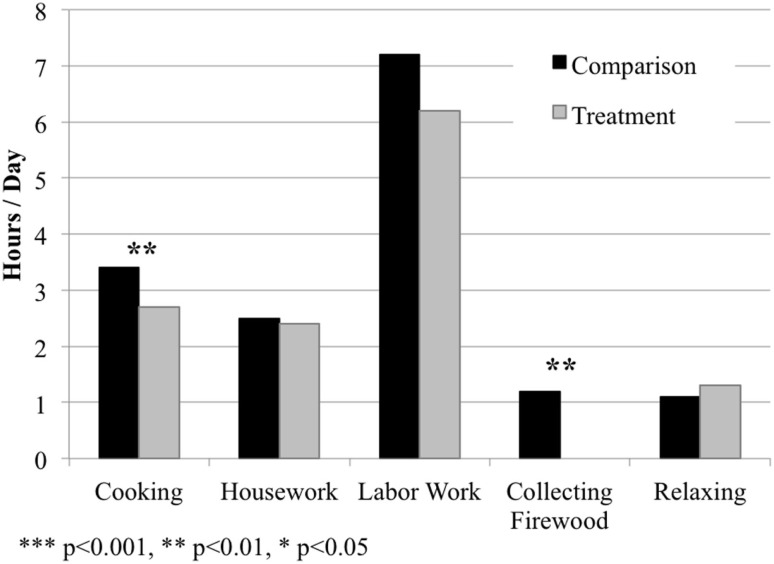
**The amount of time female household heads spent cooking, doing housework, doing labor work, collecting firewood, and relaxing per day in the treatment and comparison populations, respectively**. Values presented as mean hours spent per day on each activity. Significant differences in respondent time allocations between the two populations represented by asterisks, based on analyses from mixed models with fixed effects (see Table 3).

As with the first hypothesis, we test the relationship between a female household head’s allocations of time and ownership of a biogas cook stove using mixed models with fixed effects (Figure 4; Table 3). None of the models experienced problems with collinearity (all VIF values <1.22). The models were also run on the data subset identified as comparable through PSM, and results were consistent with the full sample analysis (Table S6 in Supplementary Material).

**Table 3:** **Mixed model with fixed effects grouped by region for female household heads’ allocations of time for five activities: cooking, housework, labor work, collecting firewood, and relaxing**. Biogas cook stove ownership reported as a binary variable with comparison households (0) and treatment households (1).

Variables	(1) Resp_cooking	(2) Resp_housework	(3) Resp_labor_work	(4) Resp_firewood	(5) Resp_relaxing
Firewood_biogas	-0.743** (0.218)	0.037 (0.209)	−0.229 (0.415)	−1.178*** (0.150)	0.219 (0.175)
Kerosene	−0.328 (0.178)	−0.768** (0.188)	0.455 (0.619)	−0.668 (0.381)	−0.512** (0.126)
Add_firewood	−0.276 (0.223)	−1.230*** (0.222)	2.267*** (0.447)	−0.269 (0.185)	−1.088*** (0.218)
Asset_index	0.267 (0.225)	0.102 (0.309)	−1.286* (0.458)	−0.125 (0.068)	0.198 (0.125)
Caste	−0.258 (0.165)	−0.139 (0.212)	0.000 (0.411)	−0.027 (0.044)	0.082 (0.113)
Religion	0.181 (0.473)	−0.751 (0.515)	−0.772 (1.006)	−0.020 (0.086)	−0.161 (0.286)
HH_size	−0.151 (0.266)	−0.311 (0.211)	0.401 (0.665)	−0.006 (0.047)	−0.329 (0.156)
Dependency_ratio	−0.071 (0.204)	0.122 (0.180)	−0.899 (0.704)	−0.021 (0.041)	0.289 (0.144)
Observations	152	152	152	152	152
Number of region	5	5	5	5	5
Adjusted *R*-squared	0.061	−0.004	0.015	0.549	0.055
RMSE	1.027	1.310	3.346	0.500	0.800

*Robust standard errors (SEs) are in parentheses*.

*** < 0.001, ** < 0.01, * < 0.05.

Results clearly show that women in households with a biogas cook stove spend significantly less time cooking and collecting firewood than women in comparison households (Table 3; Table S6 in Supplementary Material). No significant effects of owning a biogas cook stove were found on time spent doing housework, labor work, or relaxing.

Respondents from the comparison group reported spending, on average, about 0.7 more hours (~40 min) cooking and 1.2 more hours (~70 min) collecting firewood per day than respondents from the treatment group (Tables 1 and 3). This implies that women with biogas cook stoves save, on average, roughly 2 h per day just by owning these stoves. This presents an opportunity cost: while there is no guarantee of how they use the freed time, respondents with a biogas cook stove have the opportunity to re-allocate their freed time toward other activities, such as income generation, while respondents from comparison households do not have this opportunity.

Figure 4 and Table 1 further illustrate that women’s time allocations vary significantly between the treatment and comparison populations. Women with biogas cook stoves spend on average more time relaxing and less time cooking, collecting firewood, doing housework, and doing labor work than women from comparison households. However, our current findings do not provide in-depth insights or clear patterns on how women in treatment households use their extra time.

### Insights from focus group discussions

Focus group discussions provide further insight on the relationships observed in the quantitative models. First, women confirmed that households with a biogas cook stove have more diverse diets. The cooking efficiency and consumer friendly nature of the stoves were mentioned as major reasons behind this trend. Because the fuel source is now self-sufficient, burning without the constant need to maintain an adequate flame, women are able to multi-task while cooking. This allows them both to add more food items to a single dish and to diversify the type and quantity of dishes included in a meal.

Furthermore, women in the focus groups communicated a variety of other positive outcomes from owning a biogas cook stove. Specifically, they indicated that risks of collecting firewood – including thorns, snakes, and harassment from men – were considerably reduced because of the biogas cook stoves. They also reported that indoor fires – caused, for example, by a sari catching fire from the wood-burning stove – happened far less frequently with the new technology. Finally, they noted that having households in the region that used biogas cook stoves decreased total wood use in the area, leaving more available for families continuing to use wood-burning stoves.

## Discussion

Our quantitative and qualitative results indicate that households with access to a biogas cook stove have more diverse diets than households without the stoves (Figure 3; Table 2; Tables S3 and S5 in Supplementary Material). Results also indicate that women with a biogas cook stove spend less time cooking and collecting firewood (Figure 4; Table 3; Table S6 in Supplementary Material). This study therefore provides evidence of two distinct co-benefits of biogas cook stove interventions in the Bagepalli panchayat.

Several mechanisms may explain why diets are more diverse among households that own a biogas cook stove. Women described how using a biogas cook stove allowed them to multi-task while cooking, which in turn facilitated their ability to prepare a larger variety of items for a given meal. Women also described a range of further benefits from owning a biogas cook stove, including fire safety inside the house and decreased risks from collecting firewood.

Additional factors that may have contributed to the higher diet diversity among biogas cook stove users include the ability to adjust cooking temperatures and the elimination of indoor air pollution. With a wood-burning stove, it is difficult to control or predict stovetop temperatures. As a result, cooking more heat-sensitive dishes such as meat or fish can be challenging, and women using traditional fuel sources may have chosen to avoid these dishes, given the consequent spoilage risk involved. With a biogas cook stove, dishes with longer cooking times, such as rice could be cooked in a structured timeframe, thus facilitating the ease of cooking these other items. Given the difficulties and time involved in collecting firewood, dishes with longer cooking times may have also been cooked less frequently prior to owning a biogas cook stove in an effort to conserve wood.

Furthermore, several studies have illustrated that cooking with firewood produces larger amounts of particulate matter and carbon monoxide than alternative cook stoves (13–16). In line with this existing evidence (13, 18, 19, 21–24, 75), women in our study often reported concern about the impact of indoor air pollution on the health of their families. For households without a biogas cook stove, the health risk related to extensive cooking might limit their food choices. This information is purely anecdotal, as our study did not collect data specifically to examine this possible explanation. Further studies can provide more insights on these household decisions and behaviors.

It is important to note that the diet diversity of a household is mainly interpreted as an indicator of food access and can have little bearing on the nutrient intake of individuals in the household, where intra-household distribution of food plays an important role (34). Investigating effects on individual diet diversity will shed further light on the nutritional impact of biogas cook stoves.

Respondents with biogas cook stoves also spent significantly less time cooking and collecting firewood – approximately 2 h per day – than respondents relying on firewood stoves (Figure 4; Tables 1 and 3; Table S6 in Supplementary Material). This indicates an opportunity cost to women relying on wood-burning stoves. Female household heads with biogas stoves have the option to put their additional time toward, for example, income generation and relaxation. The value of these activities cannot be underestimated, especially given the strains of the desert climate, and that local employment often includes intense agricultural labor. Collectively, time saved by cooking on a biogas rather than wood stove becomes a significant additional benefit of this technological intervention.

Households in the treatment and comparison groups varied in certain socio-economic and cultural characteristics, such as caste and assets. PSM sought to account for these differences, by running the models on a subset of the study population that were equally likely to have received the treatment effect according to a collection of equally weighted variables (72). Results run on this data subset were robust with those using the full sample population.

Currently, there is a dearth of studies evaluating outcomes, such as diet diversity and time management, when considering the impact of alternative cook stoves (76, 77), and multiple reports have called for further research (76, 78–80). We hope that the promising results described in this paper will encourage other research and development initiatives to expand their set of impact indicators when studying the use of improved cook stoves. This will facilitate a more comprehensive understanding of the benefits and disadvantages of biogas and other alternative cook stove projects.

## Conclusion

In this study, we sought to evaluate two co-benefits of a biogas cook stove project in southern India. Results indicate that owning a biogas cook stove has a significant positive correlation with household diet diversity (31, 81–83). Female heads of households with a biogas cook stove also reported spending approximately two less hours cooking and collecting firewood per day. These women have the option of putting their freed time toward other activities, such as income generation and relaxation, presenting an opportunity cost to families continuing to rely on solid fuel for cooking.

Currently, low- and middle-income countries continue to extensively use solid fuels for energy intensive activities, such as cooking. Given today’s severe global health burden from openly combusting solid fuels, it is critical to explore alternative energy sources and their implications for families transitioning to new types of cook stoves (2, 4, 5, 12). While results from this analysis may be situation specific, the positive relationships observed underscore the importance of quantifying environmental, social, and economic advantages and disadvantages of household-level technological interventions, in order to better align incentives for rural development and to understand the net merit of these technologies for participating communities.

## Author Contributions

TA, RD, and RA conceived and designed the experiments; TA and SU performed the experiments; TA, RD, SW, and RR analyzed the data; all authors contributed to the writing of this paper.

## Conflict of Interest Statement

The authors declare that the research was conducted in the absence of any commercial or financial relationships that could be construed as a potential conflict of interest.

## Supplementary Material

The Supplementary Material for this article can be found online at http://journal.frontiersin.org/article/10.3389/fnut.2015.00028
