# Supplementary material for: Biogas Cook Stoves for Healthy and Sustainable Diets? A Case Study in Southern India
Source: Front Nutr. 2015 Sep 16;2:28. doi: 10.3389/fnut.2015.00028 (PMC4584993; doi:10.3389/fnut.2015.00028)
Supplement: Supplementary file 1 [file Table_1.DOCX]

***Supplementary Material***

**Biogas cook stoves for healthy and sustainable diets?
A case study in Southern India**

**Tal Lee Anderman^1^*, Ruth S. DeFries^2^, Stephen A. Wood^2,3^, Roseline Remans^3,4^, Richie Ahuja^1^, Shujayth E. Ulla^5^**

^1^ Environmental Defense Fund, San Francisco, CA, USA

^2^ Department of Ecology, Evolution, and Environmental Biology, Columbia University, New York, NY, USA

^3^ Agriculture and Food Security Center, the Earth Institute, Columbia University, New York, NY, USA

^4^ Bioversity International, Addis Ababa, Ethiopia

^5^ Department of Social Work, St. Joseph’s College, Bangalore, Karnataka, India

*** Correspondence:** Tal Lee Anderman, Environmental Defense Fund, 123 Mission Street, San Francisco, CA, 94105, USA.

Tal.anderman@gmail.com

1. **Supplementary Tables**

**Supplementary Table 1.** Descriptive statistics for villages within each study region, including their distribution of demographic, geographic, and socio-economic characteristics. Regions were designated by the geographic spread of the villages, with two to three treatment and one comparison village per region (Figure 2).

|  | **Treatment Villages** | | | | | | | | | | **Comparison Villages** | | | | |
| --- | --- | --- | --- | --- | --- | --- | --- | --- | --- | --- | --- | --- | --- | --- | --- |
| Region | **1** | | **2** | | **3** | | **4** | **5** | | | **1** | **2** | **3** | **4** | **5** |
| Village Sample Size | 12 | 12 | 9 | 26 | 8 | 17 | 11 | 13 | 21 | 18 | 13 | 7 | 14 | 12 | 12 |
| ADATS Member Households | 93 | 92 | 139 | 183 | 91 | 153 | 53 | 53 | 66 | 73 | 56 | 37 | 66 | 36 | 28 |
| Total Households | 285 | 104 | 719 | 308 | 142 | 301 | 73 | 76 | 88 | 219 | 75 | 56 | 93 | 52 | 38 |
| Kerosene Stove Users | 0 | 0 | 0 | 0 | 0 | 0 | 0 | 0 | 0 | 0 | 1 | 1 | 3 | 0 | 0 |
| Additional Firewood Stove | 0 | 0 | 0 | 0 | 0 | 0 | 0 | 0 | 0 | 2 | 0 | 0 | 0 | 0 | 0 |
| Caste  (Lower / Upper) | 7 / 4 | 0 / 9 | 1 / 7 | 4 / 18 | 6 / 1 | 5 / 6 | 7 / 3 | 6 / 4 | 4 / 8 | 1 / 4 | 11 / 1 | 2 / 5 | 12 / 2 | 3 / 9 | 10 / 2 |
| Religion  (Hindu / Muslim) | 9 / 3 | 10/0 | 7 / 2 | 18 / 8 | 7 / 0 | 15 / 2 | 11 / 0 | 12 / 0 | 20 / 0 | 12 / 5 | 13 / 0 | 5 / 2 | 14 / 0 | 12 / 0 | 12 / 0 |
| Membership in ADATS (years) | 23 | 26 | 27 | 28 | 35 | 19 | 23 | 21 | 35 | 7 | 20 | 12 | 35 | 23 | 21 |
| Distance to Major Market (km) | 14.9 | 13.4 | 28.0 | 34.0 | 25.4 | 37.8 | 28.0 | 14.4 | 10.3 | 7.8 | 17.3 | 29.1 | 24.0 | 26.5 | 21.8 |
| Household Size (average) | 6.0 | 4.8 | 5.6 | 4.1 | 7.4 | 5.6 | 4.4 | 4.7 | 5.4 | 4.3 | 5.1 | 4.2 | 4.4 | 4.3 | 5.4 |
| Dependency Ratio (average) | 0.4 | 0.3 | 0.6 | 0.2 | 0.5 | 0.4 | 0.2 | 0.5 | 0.3 | 0.4 | 0.5 | 0.5 | 0.5 | 0.3 | 0.4 |
| Asset Index (average) | 0.0 | 0.4 | 1.8 | 1.1 | 0.2 | 2.1 | -0.8 | 1.6 | 0.9 | 0.9 | -1.3 | -0.6 | -1.1 | -0.5 | -1.1 |
