# Supplementary material for: Biogas Cook Stoves for Healthy and Sustainable Diets? A Case Study in Southern India
Source: Front Nutr. 2015 Sep 16;2:28. doi: 10.3389/fnut.2015.00028 (PMC4584993; doi:10.3389/fnut.2015.00028)
Supplement: Supplementary file 2 [file Table_2.DOCX]

***Supplementary Material***

**Biogas cook stoves for healthy and sustainable diets?
A case study in Southern India**

**Tal Lee Anderman^1^*, Ruth S. DeFries^2^, Stephen A. Wood^2,3^, Roseline Remans^3,4^, Richie Ahuja^1^, Shujayth E. Ulla^5^**

^1^ Environmental Defense Fund, San Francisco, CA, USA

^2^ Department of Ecology, Evolution, and Environmental Biology, Columbia University, New York, NY, USA

^3^ Agriculture and Food Security Center, the Earth Institute, Columbia University, New York, NY, USA

^4^ Bioversity International, Addis Ababa, Ethiopia

^5^ Department of Social Work, St. Joseph’s College, Bangalore, Karnataka, India

*** Correspondence:** Tal Lee Anderman, Environmental Defense Fund, 123 Mission Street, San Francisco, CA, 94105, USA.

Tal.anderman@gmail.com

1. **Supplementary Tables**

**Supplementary Table 2.** Food groups and respective items used in the daily and weekly diet diversity scores that was also the basis of the minimum diet diversity score. Foods were categorized into 10 food groups in a distribution following the women’s diet diversity score composition as outlined by the Food and Nutrition Technical Assistance III Project and the Food and Agriculture Organization of the United Nations (55). All food items listed were used to construct the daily and weekly food variety score (41).

| **Food Groups** | **Food Items** |
| --- | --- |
| Starchy Staples | maize, millet, potato, rice, sorghum, wheat |
| Beans & Peas | bean, chickpea, lentil |
| Nuts & Seeds | peanut |
| Dairy | cheese, milk, yogurt |
| Flesh Foods | beef, chicken, fish (any), mutton, pork |
| Eggs | eggs |
| Green Leafy Vegetables | spinach |
| Vitamin A Fruit & Vegetables | carrot, chili, guava, okra, orange, papaya, sapodilla, tomato |
| Other Vegetables | cauliflower, eggplant, onion |
| Other Fruits | apple, banana, coconut, grapes, pineapple |
