# Supplementary material for: Biogas Cook Stoves for Healthy and Sustainable Diets? A Case Study in Southern India
Source: Front Nutr. 2015 Sep 16;2:28. doi: 10.3389/fnut.2015.00028 (PMC4584993; doi:10.3389/fnut.2015.00028)
Supplement: Supplementary file 3 [file Table_3.DOCX]

***Supplementary Material***

**Biogas cook stoves for healthy and sustainable diets?
A case study in Southern India**

**Tal Lee Anderman^1^*, Ruth S. DeFries^2^, Stephen A. Wood^2,3^, Roseline Remans^3,4^, Richie Ahuja^1^, Shujayth E. Ulla^5^**

^1^ Environmental Defense Fund, San Francisco, CA, USA

^2^ Department of Ecology, Evolution, and Environmental Biology, Columbia University, New York, NY, USA

^3^ Agriculture and Food Security Center, the Earth Institute, Columbia University, New York, NY, USA

^4^ Bioversity International, Addis Ababa, Ethiopia

^5^ Department of Social Work, St. Joseph’s College, Bangalore, Karnataka, India

*** Correspondence:** Tal Lee Anderman, Environmental Defense Fund, 123 Mission Street, San Francisco, CA, 94105, USA.

Tal.anderman@gmail.com

1. **Supplementary Tables**

**Supplementary Table 3.** The percentage of households consuming each of the 10 food groups in the daily and weekly diet diversity scores in the treatment and comparison populations, respectively. Significant differences in the consumption of food groups between the treatment and comparison populations are presented based on analyses from mixed models with fixed effects.

|  | **DAILY** | | **WEEKLY** | | **Significance** |
| --- | --- | --- | --- | --- | --- |
|  | **Treatment** | **Comparison** | **Treatment** | **Comparison** |  |
| Starchy Staples | 100% | 100% | 100% | 100% | *** |
| Beans & Peas | 99% | 97% | 100% | 100% |  |
| Nuts & Seeds | 10% | 2% | 96% | 88% | *** |
| Dairy | 73% | 40% | 82% | 59% | * |
| Flesh Foods | 18% | 4% | 90% | 83% | ** |
| Eggs | 2% | 2% | 70% | 59% |  |
| Green Leafy Veg. | 31% | 26% | 97% | 100% |  |
| Vit. A Fruits & Veg. | 100% | 100% | 100% | 100% | * |
| Other Vegetables | 99% | 100% | 99% | 100% |  |
| Other Fruits | 96% | 90% | 100% | 100% | ** |
| *** p<0.001, ** p<0.01, * p<0.05 | | | | | |
