# Supplementary material for: Biogas Cook Stoves for Healthy and Sustainable Diets? A Case Study in Southern India
Source: Front Nutr. 2015 Sep 16;2:28. doi: 10.3389/fnut.2015.00028 (PMC4584993; doi:10.3389/fnut.2015.00028)
Supplement: Supplementary file 4 [file Table_4.DOCX]

***Supplementary Material***

**Biogas cook stoves for healthy and sustainable diets?
A case study in Southern India**

**Tal Lee Anderman^1^*, Ruth S. DeFries^2^, Stephen A. Wood^2,3^, Roseline Remans^3,4^, Richie Ahuja^1^, Shujayth E. Ulla^5^**

^1^ Environmental Defense Fund, San Francisco, CA, USA

^2^ Department of Ecology, Evolution, and Environmental Biology, Columbia University, New York, NY, USA

^3^ Agriculture and Food Security Center, the Earth Institute, Columbia University, New York, NY, USA

^4^ Bioversity International, Addis Ababa, Ethiopia

^5^ Department of Social Work, St. Joseph’s College, Bangalore, Karnataka, India

*** Correspondence:** Tal Lee Anderman, Environmental Defense Fund, 123 Mission Street, San Francisco, CA, 94105, USA.

Tal.anderman@gmail.com

1. **Supplementary Tables**

**Supplementary Table 4.** The 20 asset indicators used to generate the asset index using a principal component analysis (59-61). The asset index derived from these indicators was used to measure wealth by aggregating household stocks with different units to generate a wealth ranking between households in the study population.

| **Asset Indicators** | |
| --- | --- |
| Own Clock or Watch | Drinking Water from Other Source |
| Own Radio | Main Source of Light Electric |
| Own Television | Flush Toilet |
| Own Sewing Machine | Pit Toilet / Latrine |
| Own Bicycle | None / Other Toilet |
| Own Motorcycle or Scooter | Own > 3.6 Acres Land |
| Own Car | Number of Rooms in Dwelling |
| Own Refrigerator | Kitchen a Separate Room |
| Drinking Water from Pump / Well | Dwelling all Low-Quality Materials |
| Drinking Water from Open Source | Dwelling all High-Quality Materials |
