# Supplementary material for: Biogas Cook Stoves for Healthy and Sustainable Diets? A Case Study in Southern India
Source: Front Nutr. 2015 Sep 16;2:28. doi: 10.3389/fnut.2015.00028 (PMC4584993; doi:10.3389/fnut.2015.00028)
Supplement: Supplementary file 5 [file Table_5.DOCX]

***Supplementary Material***

**Biogas cook stoves for healthy and sustainable diets?
A case study in Southern India**

**Tal Lee Anderman^1^*, Ruth S. DeFries^2^, Stephen A. Wood^2,3^, Roseline Remans^3,4^, Richie Ahuja^1^, Shujayth E. Ulla^5^**

^1^ Environmental Defense Fund, San Francisco, CA, USA

^2^ Department of Ecology, Evolution, and Environmental Biology, Columbia University, New York, NY, USA

^3^ Agriculture and Food Security Center, the Earth Institute, Columbia University, New York, NY, USA

^4^ Bioversity International, Addis Ababa, Ethiopia

^5^ Department of Social Work, St. Joseph’s College, Bangalore, Karnataka, India

*** Correspondence:** Tal Lee Anderman, Environmental Defense Fund, 123 Mission Street, San Francisco, CA, 94105, USA.

Tal.anderman@gmail.com

1. **Supplementary Tables**

**Supplementary Table 5.** Mixed model with fixed effects grouped by region for the daily and weekly diet diversity score and food variety score, and the minimum diet diversity score, run on a population subset selected through propensity score matching on household socio-economic characteristics. Biogas cook stove ownership reported as a binary variable with comparison households (0) and treatment households (1).

|  | (1) | (2) | (3) | (4) | (5) |  |
| --- | --- | --- | --- | --- | --- | --- |
| VARIABLES | Day_Diet_ Diversity_Score _PSM | Wk_Diet_ Diversity_Score _PSM | Day_Food_ Variety_Score_ PSM | Wk_Food_ Variety_Score_ PSM | Minimum_ Diet_Diversity_  PSM |  |
|  |  |  |  |  |  |  |
| Firewood_Biogas | 0.364*** | 0.307* | 1.560*** | 3.058** | -0.011 |  |
|  | (0.088) | (0.143) | (0.215) | (0.821) | (0.018) |  |
| Asset_Index | 0.391** | 0.238 | 1.010** | 0.333 | 0.032 |  |
|  | (0.117) | (0.180) | (0.278) | (1.001) | (0.030) |  |
| Caste | 0.360* | 0.030 | 0.621* | 0.119 | 0.052* |  |
|  | (0.135) | (0.115) | (0.256) | (0.637) | (0.022) |  |
| Religion | -0.027 | 0.275 | -0.206 | 2.448*** | 0.018 |  |
|  | (0.541) | (0.240) | (0.477) | (0.391) | (0.013) |  |
| Dist_Market | 0.490** | -0.301 | 1.526*** | -0.338 | -0.038 |  |
|  | (0.131) | (0.367) | (0.305) | (1.467) | (0.023) |  |
| HH_Size | 0.077 | -0.099 | 0.265 | -0.792 | 0.002 |  |
|  | (0.167) | (0.102) | (0.427) | (0.611) | (0.028) |  |
| Age_ADATS | 0.308 | 0.007 | 0.726 | -0.053 | 0.097* |  |
|  | (0.259) | (0.232) | (0.438) | (1.188) | (0.045) |  |
|  |  |  |  |  |  |  |
| Observations | 138 | 138 | 138 | 138 | 138 |  |
| Number of Region | 5 | 5 | 5 | 5 | 5 |  |
| Adjusted R-squared | 0.098 | 0.031 | 0.346 | 0.262 | 0.034 |  |
| RMSE | 0.910 | 0.807 | 2.087 | 3.874 | 0.141 |  |
| Robust standard errors in parentheses | | | | | | |
| *** p<0.001, ** p<0.01, * p<0.05 | | | | | | |
