# Supplementary material for: Biogas Cook Stoves for Healthy and Sustainable Diets? A Case Study in Southern India
Source: Front Nutr. 2015 Sep 16;2:28. doi: 10.3389/fnut.2015.00028 (PMC4584993; doi:10.3389/fnut.2015.00028)
Supplement: Supplementary file 6 [file Table_6.DOCX]

***Supplementary Material***

**Biogas cook stoves for healthy and sustainable diets?
A case study in Southern India**

**Tal Lee Anderman^1^*, Ruth S. DeFries^2^, Stephen A. Wood^2,3^, Roseline Remans^3,4^, Richie Ahuja^1^, Shujayth E. Ulla^5^**

^1^ Environmental Defense Fund, San Francisco, CA, USA

^2^ Department of Ecology, Evolution, and Environmental Biology, Columbia University, New York, NY, USA

^3^ Agriculture and Food Security Center, the Earth Institute, Columbia University, New York, NY, USA

^4^ Bioversity International, Addis Ababa, Ethiopia

^5^ Department of Social Work, St. Joseph’s College, Bangalore, Karnataka, India

*** Correspondence:** Tal Lee Anderman, Environmental Defense Fund, 123 Mission Street, San Francisco, CA, 94105, USA.

Tal.anderman@gmail.com

1. **Supplementary Tables**

**Supplementary Table 6.** Mixed model with fixed effects grouped by region for female household heads’ allocations of time for five activities: cooking, housework, labor work, collecting firewood, and relaxing. This model was run on a population subset selected through propensity score matching on household socio-economic characteristics. Biogas cook stove ownership reported as a binary variable with comparison households (0) and treatment households (1).

|  | (1) | (2) | (3) | (4) | (5) |  |
| --- | --- | --- | --- | --- | --- | --- |
| VARIABLES | Resp_Cooking _PSM | Resp_Housework _PSM | Resp_Labor_ Work_PSM | Resp_Firewood _PSM | Resp_Relaxing _PSM |  |
|  |  |  |  |  |  |  |
| Firewood_Biogas | -0.751** | 0.083 | -0.331 | -1.160*** | 0.176 |  |
|  | (0.235) | (0.194) | (0.363) | (0.153) | (0.169) |  |
| Asset_Index | 0.227 | 0.157 | -1.235* | -0.074 | 0.149 |  |
|  | (0.231) | (0.329) | (0.547) | (0.064) | (0.123) |  |
| Caste | -0.286 | -0.082 | 0.131 | -0.008 | 0.003 |  |
|  | (0.181) | (0.228) | (0.463) | (0.039) | (0.127) |  |
| Religion | 0.207 | -0.723 | -0.736 | -0.039 | -0.172 |  |
|  | (0.481) | (0.529) | (1.008) | (0.091) | (0.281) |  |
| HH_Size | -0.156 | -0.265 | 0.469 | 0.012 | -0.332 |  |
|  | (0.285) | (0.235) | (0.734) | (0.062) | (0.175) |  |
| Dependency_Ratio | 0.012 | -0.075 | -1.207 | -0.071 | 0.392* |  |
|  | (0.259) | (0.223) | (1.007) | (0.096) | (0.171) |  |
|  |  |  |  |  |  |  |
| Observations | 138 | 138 | 138 | 138 | 138 |  |
| Number of Region | 5 | 5 | 5 | 5 | 5 |  |
| Adjusted R-squared | 0.069 | -0.012 | 0.029 | 0.523 | 0.029 |  |
| RMSE | 1.047 | 1.346 | 3.293 | 0.492 | 0.811 |  |
| Robust standard errors in parentheses | | | | | | |
| *** p<0.001, ** p<0.01, * p<0.05 | | | | | | |
