# Supplementary material for: Biogas Cook Stoves for Healthy and Sustainable Diets? A Case Study in Southern India
Source: Front Nutr. 2015 Sep 16;2:28. doi: 10.3389/fnut.2015.00028 (PMC4584993; doi:10.3389/fnut.2015.00028)
Supplement: Supplementary file 8 [file Survey.PDF]

Low-Carbon Development Project in Rural India Survey

Assessing co-benefits for food and nutritional security, respiratory health, and income

Section I. Survey Information

Socio-Economic Survey Number

I.i Date of Interview   m m | d d | y y

I.ii Interviewer

I.iii Household Head

I.iv Member Code

I.v Notes

Section II. Household Information

II.i Taluk

II.ii GP_name

II.iii Village

II.vi Language

II.vii Religion

II.viii Household Size

Household Member Information

	a) Name	b) Relationship to HH Head	c) Age
II.xi			
II.xii			
II.xiii			
II.xiv			
II.xv			
II.xvi			

II.xvii Husband Occupation

II.xviii Do you currently use a biogas fueled cooking stove? Yes | No

a If so, when was the unit established? Month | Day | Year

II.xix Do you have a Television? | Yes | No |

II.xx Notes

Section III. Dietary Diversity

III. How many times per day, week, or month does your household eat the following foods?

Food Source and Type		a) Yesterday	b) Times	c) per [ ]	d) Notes
**STAPLES (Grains, Tubers, Legumes)**					
III.i	Rice				
III.ii	Ragi				
III.iii	Wheat				
III.iv	Sorghum (Jowar)				
III.v	Maize				
III.vi	Chickpeas				
III.vii	Lentils (Daal)				
III.viii	Groundnuts				
III.ix	Other Cereal/Pulse				
List:					
**VEGETABLES**					
III.x	Beans				
III.xi	Carrots				
III.xii	Tomatoes				
III.xiii	Brinja				
III.xiv	Onion				
III.xv	Spinach				
III.xvi	Potatoes				
III.xvii	Okra (Lady Finger)				
III.xviii	Cauliflower				
III.xx	Chillies				
**FRUIT**					

III.xxi	Banana				
III.xxii	Guava				
III.xxiii	Grapes				
III.xxiv	Orange				
III.xxv	Apple				
III.xxvi	Coconut				
III.xxvii	Pineapple				
III.xxviii	Chiku				
III.xxix	Papaya				
**MEAT / FISH / EGGS**					
III.xxx	Eggs (number)				
III.xxxi	Chicken				
III.xxxii	Fish				
III.xxxiii	Beef (cow)				
III.xxxiv	Pork (pig)				
III.xxxv	Mutton (sheep)				
**DAIRY**					
III.xxxvi	Milk				
III.xxxvii	Yogurt				
III.xxxviii	Paneer (Cheese)				
III.xxxix	Other Milk Product				
List:					
**OTHER (SUGAR, OIL, SNACKS, BEVERAGES)**					
III.xxxx	Tea, Coffee, Chai				
III.xxxxi	Soda (Dosa) / Cola				
III.xxxxii	Ice cream				
III.xxxxiii	Biscuits				

III.xxxxiv Note: List below any additional foods or unusual circumstances (no salt, sugar, or spices)

III.xxxxv How many meals did your household consume yesterday?

III.xxxxvi Does your family cook new (types of) foods with the bio-gas cookstove? Yes No

a If yes, what kinds of foods?

III.xxxxvii In these months, was there a day in which your household did not have enough food?

Jan	Feb	Mar	April	May	Jun	July	Aug	Sept	Oct	Nov	Dec

Note: Record MIHFS

III.xxxxviii Notes

Section IV. Female Allocations of Time

RESPONDANT

How many hours did [respondant] spend engaging in the following activities?

Did they receive any payment (monetary, gift, otherwise) for this activity?

	Activity	a) Time	b) Hr/day	c) Compensation?	d) Notes
IV.i	Cooking				
IV.ii	Housework (clean)				
IV.iii	Labor work				
	List:				
IV.iv	Relaxing (TV etc.)				
IV.v	Socializing				
IV.vi	Other				
	List:				

WOMAN # 2

How many hours did [Woman #2] spend engaging in the following activities?

Did they receive any payment (monetary, gift, otherwise) for this activity?

	Activity	a) Time	b) Hr/day	c) Compensation?	d) Notes
IV.vii	Cooking				
IV.viii	Housework (clean)				
IV.ix	Labor work				

	List:				
IV.x	Relaxing (TV etc.)				
IV.xi	Socializing				
IV.xii	Other				
	List:				

WOMAN # 3

How many hours did [Woman #3] spend engaging in the following activities?

Did they receive any payment (monetary, gift, otherwise) for this activity?

	Activity	a) Time	b) Hr/day	c) Compensation?	d) Notes
IV.xiii	Cooking				
IV.xiv	Housework (clean)				
IV.xv	Labor work				
	List:				
IV.xvi	Relaxing (TV etc.)				
IV.xvii	Socializing				
IV.xviii	Other				
	List:				

IV.xix Notes

Section V. Children Allocations of Time

V.i Name of Child #1:

V.ii What is the highest level of schooling [CHILD 1] attended?

How many hours did [Child #1] spend engaging in the following activities?

Did they receive any payment (monetary, gift, otherwise) for this activity?

	Activity	a) Time	b) Hr/day	c) Compensation?	d) Notes
V.iii	School				

V.iv	Homework				
V.v	Helping with house				
V.vi	Labor Work				
	List:				
V.vii	Playing				
V.viii	Other				
	List:				

V.ix Name of Child #2:

V.x What is the highest level of schooling [CHILD 2] attended?

How many hours did [Child #2] spend engaging in the following activities?
Did they receive any payment (monetary, gift, otherwise) for this activity?

	Activity	a) Time	b) Hr/day	c) Compensation?	d) Notes
V.xi	School				
V.xii	Homework				
V.xiii	Helping with house				
V.xiv	Labor Work				
	List:				
V.xv	Playing				
V.xvi	Other				
	List:				

V.xvii Name of Child #3:

V.xiii What is the highest level of schooling [CHILD 3] attended?

How many hours did [Child #3] spend engaging in the following activities?
Did they receive any payment (monetary, gift, otherwise) for this activity?

	Activity	a) Time	b) Hr/day	c) Compensation?	d) Notes
V.xiv	School				
V.xv	Homework				

	Activity	a) Time	b) Hr/day	c) Compensation?	d) Notes
V.xvi	Helping with house				
V.xvii	Labor Work				
	List:				
V.xviii	Playing				
V.xix	Other				
	List:				

V.xx Name of Child #4:

V.xxi What is the highest level of schooling [CHILD 4] attended?

How many hours did [Child #4] spend engaging in the following activities?

Did they receive any payment (monetary, gift, otherwise) for this activity?

	Activity	a) Time	b) Hr/day	c) Compensation?	d) Notes
V.xxii	School				
V.xxiii	Homework				
V.xxiv	Helping with house				
V.xxv	Labor Work				
	List:				
V.xxvi	Playing				
V.xxvii	Other				
	List:				

V.xxviii Notes

Section VI. Respiratory Health

[Respondant] Respiratory Health

		a) Headache	b Asthma/Cough	c) Lung Disease	d) TB
VI.ii	Suffered last month? (Dec 2012)				
VI.iii	Does it keep her from working?				

VI.iv	Does it require a doctors visit?				
VI.v	Does she take medication for this?				
VI.vi	Estimated Cost of the Problem?				
VI.vii	Unit of cost (per visit, week, month)				

[OTHER WOMEN (plural)] Respiratory Health

		a) Headache	b Asthma/Cough	c) Lung Disease	d) TB
VI.ix	Suffered last month? (Dec 2012)				
VI.x	Does it keep her from working?				
VI.xi	Does it require a doctors visit?				
VI.xii	Does she take medication for this?				
VI.xiii	Estimated Cost of the Problem?				
VI.xiv	Unit of cost (per visit, week, month)				

[ALL CHILDREN] Respiratory Health

		a) Headache	b Asthma/Cough	c) Lung Disease	d) TB
VI.xv	Suffered last month? (Dec 2012)				
VI.xvi	Does it keep her from working?				
VI.xvii	Does it require a doctors visit?				
VI.xviii	Does she take medication for this?				
VI.xix	Estimated Cost of the Problem?				
VI.xx	Unit of cost (per visit, week, month)				

V.xxii Notes (e.g. of diff health status for specific children, women)	

Low Carbon Development Project in Rural India Survey

Section VII. ASSET INFORMATION Survey Number

Village:	Household Head:		
Taluk:	Date:		

VII.i Do you own or have the following features in your home?

Clock or Watch	Bicycle	Drinking Water From Pump/Well	Flush Toilet	Number of Rooms in Dwelling
Radio	Motorcycle or Scooter	Drinking Water From Open Source	Pit Toilet/Latrine	Kitchen a Separate Room
Television	Car	Drinking Water From Other Source	None/Other Toilet	Dwelling all Low-Quality Materials
Sewing Machine	Refrigerator	Main Source of Light Electric	Own > 3.6 Acres Land	Dwelling all High-Quality Materials

Section VII. ASSET INFORMATION Survey Number

Village:	Household Head:		
Taluk:	Date:		

VII.i Do you own or have the following features in your home?

Clock or Watch	Bicycle	Drinking Water From Pump/Well	Flush Toilet	Number of Rooms in Dwelling
Radio	Motorcycle or Scooter	Drinking Water From Open Source	Pit Toilet/Latrine	Kitchen a Separate Room
Television	Car	Drinking Water From Other Source	None/Other Toilet	Dwelling all Low-Quality Materials
Sewing Machine	Refrigerator	Main Source of Light Electric	Own > 3.6 Acres Land	Dwelling all High-Quality Materials

Section VII. ASSET INFORMATION Survey Number

Village:	Household Head:		
Taluk:	Date:		

VII.i Do you own or have the following features in your home?

Clock or Watch	Bicycle	Drinking Water From Pump/Well	Flush Toilet	Number of Rooms in Dwelling
Radio	Motorcycle or Scooter	Drinking Water From Open Source	Pit Toilet/Latrine	Kitchen a Separate Room
Television	Car	Drinking Water From Other Source	None/Other Toilet	Dwelling all Low-Quality Materials
Sewing Machine	Refrigerator	Main Source of Light Electric	Own > 3.6 Acres Land	Dwelling all High-Quality Materials
